# A murine model of peanut-allergic asthma

**DOI:** 10.3389/falgy.2024.1378877

**Published:** 2024-04-25

**Authors:** Marta Paolucci, Nathalie Antz, Valentine Homère, Isabel Kolm, Thomas M. Kündig, Pål Johansen

**Affiliations:** ^1^ Department of Dermatology, University of Zurich, Zurich, Switzerland; ^2^ Department of Dermatology, University Hospital Zurich, Zurich, Switzerland

**Keywords:** mouse model, peanut allergy, asthma, histology, cytokines

## Abstract

**Objectives:**

Peanut allergy is an IgE-mediated food allergy that is associated with asthma in certain patients. With increasing prevalence, its great impact on the quality of life, and a lack of treatment options, the need for new therapy options is a given. Hence, models for research and development are required. This study aimed to establish a murine model of allergic airway inflammation induced by peanut allergens.

**Methods:**

C3H mice were sensitised by intraperitoneal injections of peanut allergen extract and challenged by an intranasal application of the same extract. The assessment of airway inflammation involved the analysis of immune cells in the bronchoalveolar lavage fluid as measured by flow cytometry. Inflammatory reactions in the lung tissue were also studied by histology and quantitative PCR. Moreover, peanut-specific immune responses were studied after re-stimulation of spleen cells *in vitro*.

**Results:**

Sensitisation led to allergen-specific IgE, IgA, and IgG1 seroconversion. Subsequent nasal exposure led to allergic airway inflammation as manifested by structural changes such as bronchial smooth muscle hypertrophy, mucus cell hyperplasia, infiltration of eosinophil cells and T cells, as well as an upregulation of genes expressing IL-4, IL-5, IL-13, and IFN-γ. Upon re-stimulation of splenocytes with peanut allergen, increased secretion of both T-helper type 2 (Th2) and Th1 cytokines was observed.

**Conclusion:**

We successfully established a peanut-associated asthma model that exhibited many features characteristic of airway inflammation in human patients with allergic asthma. The model holds potential as a tool for investigating novel therapeutic approaches aimed at preventing the development of allergic asthma.

## Introduction

Food allergy is described as an excessive response of the immune system to otherwise harmless food proteins (1), occasionally resulting in respiratory symptoms upon allergen exposure (2). Individuals with coexisting food allergy and asthma often experience more severe asthmatic reactions (3). Among food allergens, peanuts are known to induce the most severe allergic manifestations, often leading to systemic responses with a high risk of anaphylaxis. The dominant allergen trigger in peanut allergy is the storage protein Ara h 2 (4). Unlike other allergies that may be outgrown with age, peanut allergy tends to persist, significantly impacting patients throughout their lives (5–7).

Patients with peanut allergy typically experience intermittent airway obstruction with chest tightness, wheezing, and dyspnoea (8). Food allergy-associated asthma varies in severity, ranging from mild to severe, and may potentially end up in fatal anaphylactic shock with respiratory arrest (2). Asthma is mediated by allergen-specific IgE antibodies and attributed to an inflammation of the lower respiratory tract with increased mucus production, swelling, bronchial constriction, and infiltration of immune cells such as lymphocytes, eosinophils, and mast cells in lung tissue (8, 9). Chronic food-allergic asthma results in structural changes in the lung with airway wall remodelling and the thickening of smooth muscle cells and of the basement membrane (10).

The current treatments for food allergy symptoms are limited to allergen avoidance, bronchodilators inhalation, as well as an epinephrine autoinjector in case of anaphylaxis (5). These measures therefore only provide a short-term symptomatic relief and do not serve as a curative treatment. As the prevalence of food allergy and asthma rises globally, these conditions pose a growing health concern (11, 12).

The pathophysiological process of peanut-allergic asthma involves a type I hypersensitivity reaction (13). Following an initial peanut allergen exposure, dendritic cells (DCs) present the allergen to T-helper type 2 (Th2) lymphocytes. This induces the release of cytokines such as IL-4 and IL-13 that drive B cells to switch their production of immunoglobulin to the IgE isotype (13, 14). The formation of allergen-specific IgE antibodies results in sensitisation and disrupted tolerance (4, 15). Newly produced allergen-specific IgE antibodies then bind high affinity FcεRI on mast cells (16). Upon subsequent allergen exposure, the allergen crosslinks bound IgE antibodies, induces mast cells to de-granulate (15), releasing into the bloodstream vasoactive substances such as histamine, leukotrienes, and tryptase that contribute to early- and late-phase allergic reactions (13, 14, 17). The Th2-like cell responses and cytokines, including IL-4, IL-5, IL-13, and TNF-α, play a central role in the immunological pathway in patients with allergic asthma (18). IL-4, in addition to its role in the IgE isotype switch in B cells, contributes to the differentiation of the Th2 cells, while supressing a Th1 cell response (14, 19). An essential feature of allergic asthma is the increased migration of eosinophils into the sputum, stimulated and recruited by IL-5 (14, 19–21). Eosinophilia has demonstrated to be responsible for bronchial hyper-responsiveness, and for increased mucus production in allergy-induced asthmatic airways (14, 22). IL-13 acts similarly to IL-4 but also plays an additional role in the regulation of mucus production (14). TNF-α stimulates airway epithelial cells and increases the number of cell adhesion molecules, facilitating migration of lymphocytes to the site of inflammation (14, 19). The characteristic Th2 secretion pattern ultimately induces chronic tissue inflammation in the lungs, attracting more inflammatory effector cells (14, 15), thus establishing a positive feedback (9).

Although multiple murine models of food allergies exist, they do not assimilate the complete human allergic pathology and clinical manifestations (23). Our study aims to establish a comprehensive mouse model of peanut allergy-induced asthma. Because mice do not spontaneously develop allergies, this has to be artificially induced, considering factors such as sensitisation protocols, allergen form, adjuvants, and mouse strains (23). Building upon a previously published peanut allergy mouse model (24) characterised by elevated allergen-specific IgE titres and a Th2-like cytokine profile, our goal is to create a robust model. The successful development of such a model could serve as a standardised tool for evaluating novel therapeutic approaches against peanut allergy. Furthermore, this groundwork may be adaptable to other allergic disorders, including conditions like asthma triggered by respiratory allergens such as tree pollen or house dust mites.

## Material and methods

### Mice and ethics

Female C3H mice were purchased from Envigo (Horst, the Netherlands). The mice were kept in a pathogen-free and animal-friendly environment at ca. 21°C and with a 12–12 h light–dark cycle at the Laboratory Animal Services Centre, University of Zurich. They had open access to water and chow, and were kept in unit cages of five. The mice were not further randomised. Housing, care, and treatment of the mice were carried out according to good animal practices, following the Swiss guidelines. The mice entered the experiments at ca. 6 weeks of age. The experiments were approved by the ethical review board and Cantonal Veterinary Office of Zurich (license ZH 147/2021).

### Peanut allergen sensitisation and induction of allergic airway inflammation

**Figure 1:**
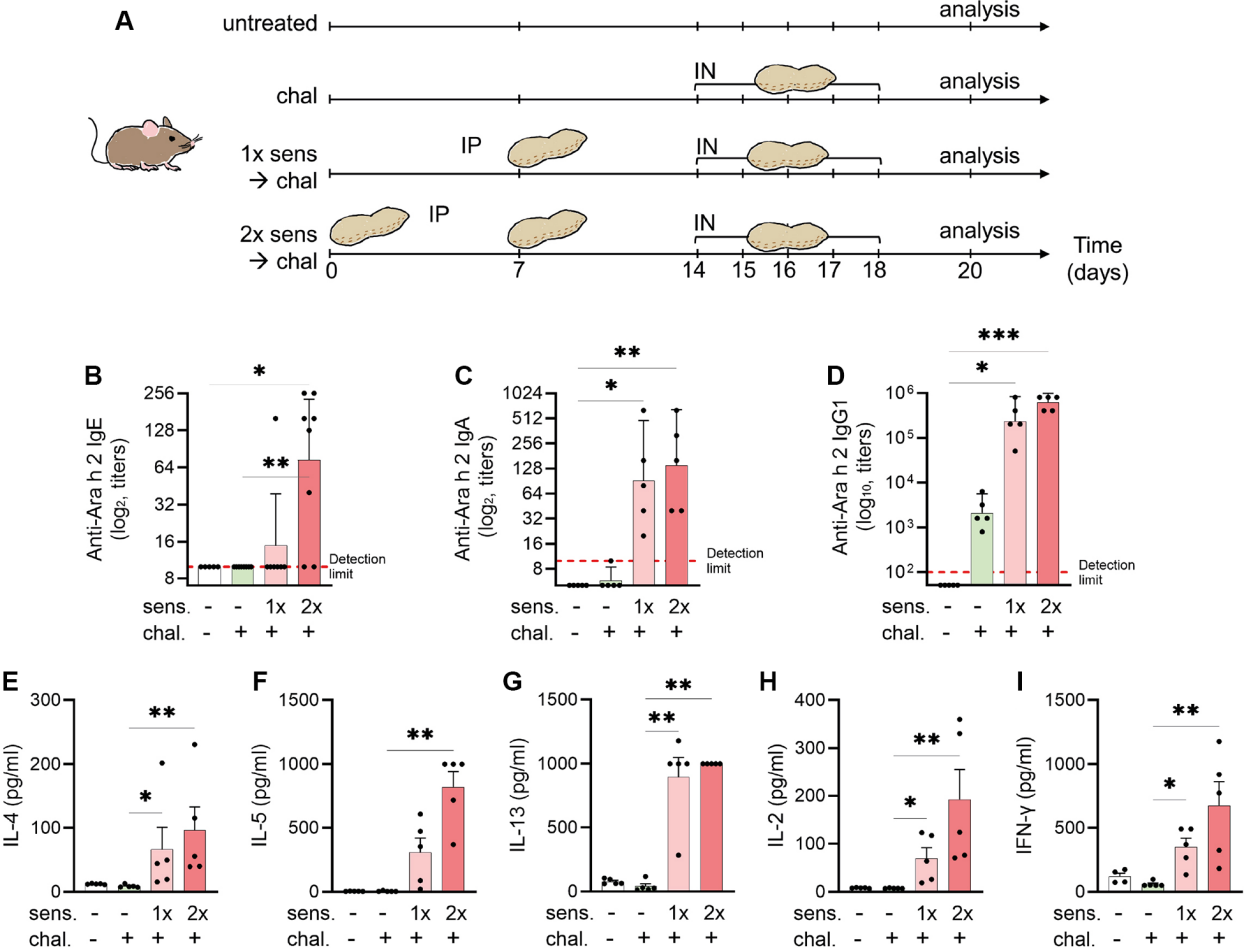
Experimental setup for a murine model of peanut-allergic asthma and detection of allergen-specific serum antibodies and cytokines. (**A**) C3H mice were sensitised (sens) IP and challenged (chal) IN for analysis of allergic airway inflammation (*n* = 5 per group). Sensitisation with peanut extract was done on days 0 and 7 (sens 2×) or day 7 only (sens 1×). The challenge with peanut extract was performed on days 14–18. Euthanasia and analysis followed on day 20. Blood sera were analysed by ELISA for the presence of Ara-h-2-specific IgE (**B**), IgA (**C**), and IgG1 (**D**) antibodies. Antibodies titres are shown as geometric mean with 95% CI. (**E**–**I**) Splenocytes were re-stimulated with peanut allergen extract and the supernatants were analysed for the content of IL-4 (**E**), IL-5 (**F**), IL-13 (**G**), IL-2 (**H**), and IFN-γ (**I**) by ELISA and illustrated as mean ± SEM. Statistical analyses were performed using the Kruskal–Wallis with Dunn test. *p < 0.05; **p < 0.01. All data are representative of the two experiments.

The mice were sensitised by intraperitoneal (IP) injections of 4.2 μg of a peanut allergen extract skin-prick-test solution (Allergopharma, Reinbek, Germany) mixed with 150 μg aluminium hydroxide (alum) adjuvant from InvivoGen (San Diego, CA, USA) and phosphate buffered saline (PBS) constituting a total of 50 μl as previously described in a peanut allergen model of anaphylaxis (24). The allergen–alum mixture was left at room temperature one hour before injection. The IP sensitisation was performed once or twice with a seven days interval. One week after the last sensitisation, the mice were challenged for five consecutive days by intranasal (IN) application of 42 μg of a purified peanut allergen extract (Inbio, Cardiff, UK) mixed with PBS in a total volume of 50 μl. The control groups of mice were kept non-sensitised and non-challenged as baseline control or kept non-sensitised but challenged. Before each challenge dose, the mice were transiently anesthetised with 2% isoflurane. Forty-eight hours after the last challenge, the mice were euthanised by IP injection of a xylazine (90 μg, Rompun-2%, Bayer Health Care, Leverkusen, Germany) and ketamine (1,800 μg, Ketasol-100, Graeub AG, Bern, Switzerland) mixture. Spleen, lung, blood, and bronchoalveolar lavage fluid (BALF) were collected for further experiments and analysis. An experimental scheme is illustrated in Figure 1A.

### Splenocytes isolation for *in vitro* stimulation

Excised spleens were aseptically processed into a single cell suspension, passed through a cell strainer and washed with PBS and foetal calf serum (FCS, 2%). The samples were centrifuged and the pellet treated with red blood cell lysis buffer (Thermo Fisher Scientific, Waltham, MA, USA) and washed twice with PBS. The cells were then resuspended in RPMI 1640 cell culture medium supplemented with 10% FCS and 1% L-glutamine. The cells were counted with a MoxiFlow (Orflo, Idaho, USA) and plated on round-bottom 96-well plates at 0.5 × 10^6^ cells in a volume of 100 μl and in triplicates. Peanut allergen extract was added at 50 μg/ml and the plates incubated at 37°C with 5% CO_2_ for 24 or 96 h. The supernatants were frozen at −20°C for a later analysis of cytokines by enzyme-linked immunosorbent assay (ELISA).

### Enzyme-linked immunosorbent assay

We applied the ELISA for detection of cytokines in the supernatant of cultured splenocytes and for the detection of Ara-h-2-specific antibodies in murine blood serum. The ELISAs were performed on 96-well Maxisorb plates. The absorbance was read at 450 nm with a Spark microplate reader (Tecan, Männedorf, Switzerland).

For the detection of peanut-specific IgE, the plates were coated with anti-mouse-IgE (BioRad, California, USA) in carbonate buffer at pH 9.6. After blocking for 1 h with 5% skimmed milk in PBS with 0.05% polysorbate 20, serial dilutions of the mouse serum were added. Next, biotin natural Ara h 2 (Inbio) was added, and the ELISA was further developed by incubation of streptavidin-conjugated horse-radish peroxidase (BioLegend, San Diego, CA, USA) followed by tetramethylbenzidine substrate (eBioscience, as purchased from Thermo Fisher Scientific, Basel, Switzerland). The colorimetric enzyme reaction was stopped with 2N sulphuric acid. Each incubation step was followed by washing the plates thrice with 0.05% polysorbate 20 in PBS.

To measure Ara-h-2-specific IgA and IgG1 levels, the plates were coated with 1 µg/ml of purified natural Ara h 2 (Inbio), and serially diluted mouse serum was added. Detection was carried out using biotinylated biotin rat anti-mouse IgA (BD Pharmingen, Franklin Lakes, USA) and rat goat-mouse IgG1 antibody (Abcam, Cambridge, UK), and the assay was developed with HRP-conjugated streptavidin and TMB, following the same procedure as described previously.

To determine the antibody titre, we measured the optical density in sera from naïve mice. The seroconversion threshold was set by calculating the mean plus 3 standard deviations above the mean using the naive mouse serum data. The anti-Ara h 2 IgE, IgA, and IgG1 titres were defined as the reciprocal final dilution at which OD were higher than the threshold.

For cytokine detection in a supernatant of splenocytes cultures, kits from eBioscience were applied. Briefly, the plates were coated with anti-cytokine capture antibodies in carbonate buffer. The culture samples and controls were diluted 1:1 in test diluent following the manufacturer's protocol. The tested cytokines included IL-2 (24-h cultures) and IL-4, IL-5, IL-13, and IFN-γ (96-h cultures). Plotting of standard curves and equations for the best linear fit were then applied to calculate the concentration levels of cytokines.

### Flow cytometry of bronchoalveolar lavage fluid

To collect the BALF from mice, a 22-gauge needle was used as a catheter and gently inserted into the trachea of a supine mouse. A cold solution of PBS (1 ml) was injected and aspirated to harvest cellular contents from the lung lumen of mice for subsequent *ex vivo* analysis. The process was repeated twice. The processing of BALF for flow cytometry was performed on ice. The total BALF volume was measured, filtered through sterile gauze, and then centrifuged at 4°C and 1,500 rpm for 5 min before being resuspended in cold PBS. After cell counting with MoxiFlow, the samples were once again centrifuged and the pellet resuspended in PBS with 2% FCS (FACS buffer) on a 96-well plate. The Fc receptors on cells were blocked by incubating the cells with anti-CD16/32 (eBioscience) on ice for 15 min. The cell staining was performed by incubating cells with a cocktail of fluorescently labelled antibodies (1:100) in the dark for 30 min for identification of T cells, B cells, macrophages, dendritic cells, neutrophils, and eosinophil cells. The following fluorescent antibodies were used: CD3e-FITC, CD19-FITC, CD11c-PE-Cy7, CD11b-PE, LY-6G-PerCP-Cy5.5, MHC II-APC, and SiglecF-BV421. All antibodies were purchased from BD Biosciences (Allschwil, Switzerland) or eBioscience. The cells were fixed in 1% paraformaldehyde and on ice for 30 min, washed in FACS buffer, and kept in the dark before acquisition on flow cytometer LSR II Fortessa 4l (BD, Heidelberg, Germany) operating on BD FACS DIVA software. The obtained data were analysed with Flow Jo 10 software (FlowJo LLC, Ashland, OR).

### Histology

Mice lung tissue was harvested, fixed in 4% formalin in PBS overnight, dehydrated, and embedded in paraffin. Histological sections of 5 μm thickness were cut using an HM 325 Rotary Microtome (Thermo Fisher Scientific). The sections were further stained with haematoxylin and eosin (H&E) and Periodic acid–Schiff (PAS) using a standardised in-house procedure. The stained sections were scanned with Zeiss Axio Scan.Z1 Slidescanner (Zeiss, Oberkochen, Germany) and Akoya Vectra Polaris (Akoya Biosciences, Marlborough, MA, USA). QuPath (version 0.5.1) was used for the H&E staining quantification.

### Quantitative polymerase chain reaction

Cytokine gene expression in the lungs was measured using a two-step quantitative polymerase chain reaction (qPCR). Tissue samples were lysed and homogenised with TissueLyser II (Qiagen, Venlo, the Netherlands) and TRIzol Reagent (Invitrogen). Following the manufacturer's protocol, total RNA was then extracted in a first step. RNA purity and concentration were assessed with a Nanodrop (Witec AG, Sursee, Switzerland). The RNA was stored at −70°C until reverse transcription into cDNA using the RevertAid First Strand cDNA Synthesis Kit (Thermo Fisher Scientific). For qPCR experiments, cDNA samples were diluted to 1:10 and measured on a LightCycler480 (Roche, Basel, Switzerland) using specific PCR Primers (Table 1) and FastStart Essential DNA Green Master (Roche Diagnostics, Rotkreuz, Switzerland) for amplification and detection. Peptidylprolyl isomerase A (PPIA) was selected as the housekeeping gene as described (25) and as confirmed in pre-tests with NormFinder (MOMA, Aarhus, Denmark), having the lowest inter and intra group differences in gene expression (25, 26). The expression levels of genes of interest were then normalised to PPIA. Up to 50 PCR cycles were conducted, and the cycle threshold value (Ct) was determined when the fluorescent signal of amplified DNA was first measured above the background fluorescence. The test samples were measured as triplicates for every gene of interest. The mean Ct value of each set of triplicate values was inserted in the double delta Ct formula (2^-ΔΔCt^) and used for the calculation of the fold change of gene expression as compared with PPIA expression.

**Table 1:** Murine genes and primer sequences used in qPCR analysis.

Genes	Forward primer (3′-5′)	Reverse primer (5′-3′)
PPIA	CCC ACC GTG TTC TTC GAC AT	CCA GTG CTC AGA GCT CGA AA
IL-4	CCA TAT CCA CGG ATG CGA CA	AAG CCC GAA AGA GTC TCT GC
IL-5	CGT GGG GGT ACT GTG GAA AT	AAT CCA GGA ACT GCC TCG TC
IL-13	TGC CAT CTA CAG GAC CCA GA	CTC ATT AGA AGG GGC CGT GG
IFN-γ	ACA GCA AGG CGA AAA AGG ATG	TGG TGG ACC ACT CGG ATG A
TNF-α	GCA CCT CAG GGA AGA GTC TG	CTG GCA CAG TGA CCT GGA CT

### Statistical analysis

The acquired data from all experiments were statistically analysed using GraphPad Prism software (version 9, GraphPad, La Jolla, CA, USA). The descriptive statistics were calculated and expressed as means, and standard error of the means were calculated. Antibody titres are presented as geometric means with 95% confidence intervals. All data were defined as non-parametric and analysed using Kruskal–Wallis tests, followed by Dunn's *post-hoc* tests for multiple comparisons. The significance level was set at 95%, with *p* < 0.05.

## Results

### Allergen-specific IgE seroconversion in mice after sensitisation with peanut extract

Because peanut allergy and allergic asthma are considered IgE mediated, we measured peanut-allergen-specific (Ara h 2) IgE antibodies in mice sera by ELISA. The mice that were not sensitised had no detectable Ara-h-2-specific IgE titre (Figure 1B). A single IP sensitisation with peanut allergen extract did not trigger IgE seroconversion (geometric mean 9.4), but two injections of the allergen extract caused a statistically significant (*p* < 0.05) induction of allergen-specific IgE antibodies (geometric mean 73.6), and seroconversion was detected in all mice. Similarly, in the assessment of IgA levels (Figure 1C), the primary isotype associated with mucosal immunity, there was a notable increase in anti-Ara h 2 titres among mice sensitised once (geometric mean 91.90) and twice (geometric mean 139.3) to peanut, compared with non-sensitised controls (geometric mean 5). In addition, we evaluated IgG1 titres (Figure 1D) targeting the key peanut allergen Ara h 2. The mice sensitised either once (geometric mean 235,253) or twice (geometric mean 620,838) prior to the challenge exhibited markedly elevated titres compared with the non-sensitised mice (geometric mean 50).

### Increased secretion of Th2- and Th1-like cytokines in mice with peanut-allergen sensitisation

After the peanut sensitisation and intranasal challenge of mice, the animals were euthanised and the harvested spleen cells were cultured *in vitro* with peanut protein extract. When measuring the cytokine secretion from non-sensitised mice, we observed that IL-4, IL-5, IL-13, IL-2, and IFN-γ were hardly detectable, independent of the mice receiving an intranasal challenge with peanut allergen or not (Figures 1E–I). By contrast, when the mice were sensitised prior to the intranasal challenge, a clear increase in secretion of all tested cytokines was observed. While there was a tendency of more cytokine secretion in cells from mice sensitised by two IP injections of peanut allergen extract, the differences between mice with single or double sensitisation were not statistically significant. The increased cytokine levels in cell cultures from mice that were sensitised twice compared with challenged-only were significant for all cytokines (*p* < 0.01). The Th2-like cytokines, IL-4 (Figure 1E), IL-5 (Figure 1F), and IL-13 (Figure 1G), in sensitised mice showed a 10–100-fold increase as compared with non-sensitised but challenged mice. The measurement of IL-2 (Figure 1H), which is a facultative Th2/Th1-like cytokine, was also significantly increased in sensitised mice as compared with challenged-only mice and the Th1-like cytokine. Interestingly, also the Th1-like cytokine IFN-γ (Figure 1I) was increased in sensitised mice.

### Infiltration of eosinophils and T cells in bronchoalveolar lavage fluid of peanut-allergen-sensitised mice

**Figure 2:**
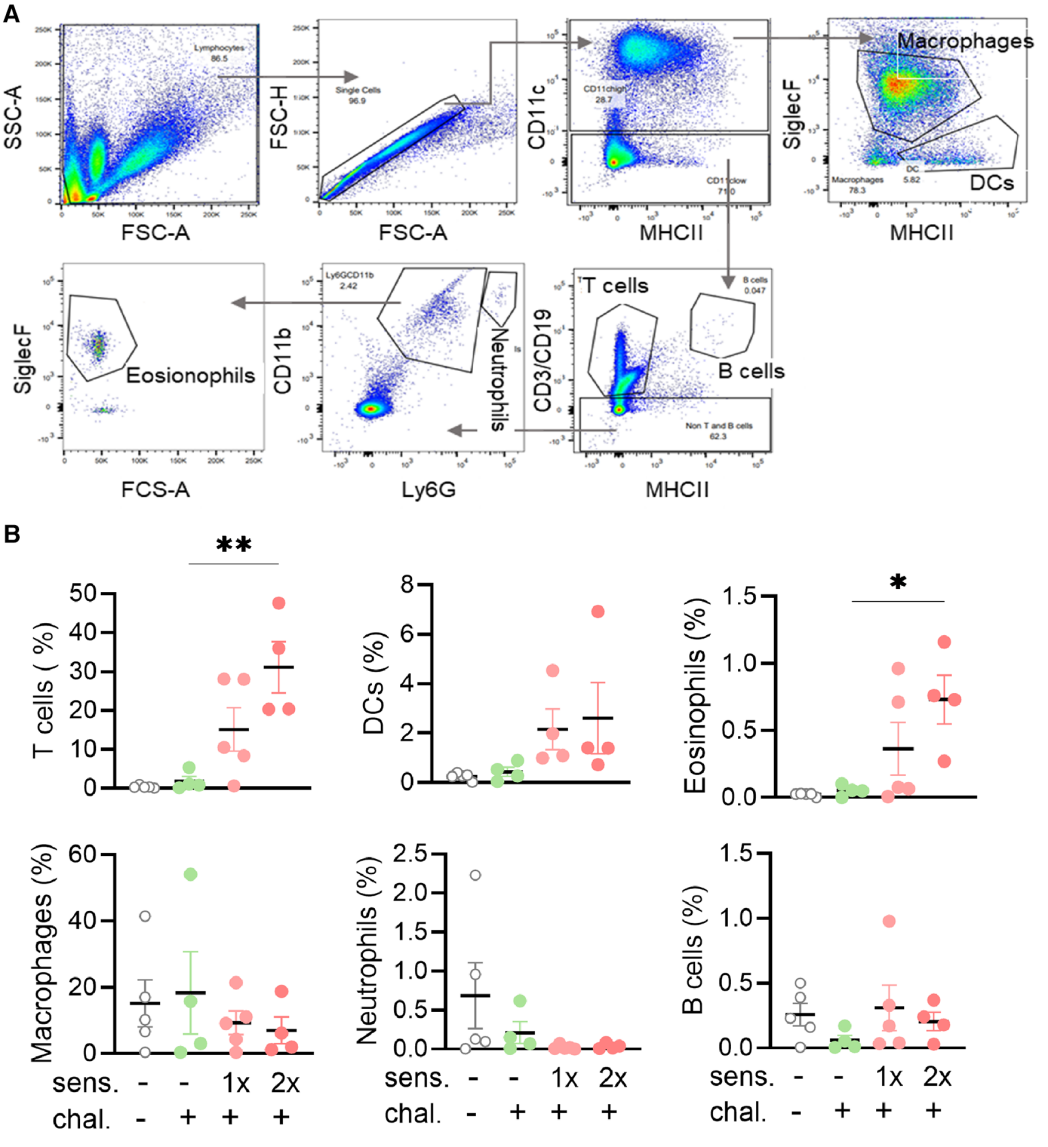
Flow cytometry gating strategy with percentages and absolute cell numbers of various immune cells found in BALF. (**A**) BALF was isolated and the leukocytes and single cells thereof identified with side scatter (SSC-A) and forward scatter area (FSC-A/FSC-H) and height (FSC-H). Sequential gating for MHC II, CD11b, CD11c, Ly6G, and SiglecF led to the identification of six leukocyte subpopulations. (**B**) BALF samples were analysed for leukocyte phenotypes. The percentages of T cells, B cells, DCs, macrophages, neutrophils, and eosinophils groups are shown as mean ± SEM. Data were analysed using the Kruskal–Wallis with Dunn's test. *: *p* < 0.05, **: *p* < 0.01. sens, sensitised; chal, challenged.

To further analyse the phenotype of mice sensitised and intranasally challenged with peanut allergen extract, a BAL was performed and the BALF samples were analysed by flow cytometry for the content of leukocytes. Different cell populations were distinguished based on their expressed surface antigens (Figure 2A). The percentage of CD3^+^ T cells in the BALF was significantly higher (*p* < 0.01) in the double-sensitised mice than in the non-sensitised challenged mice (Figure 2B). A similar trend was also noted for CD11c^+^ dendritic cells and especially for CD11b^+^ SiglecF^+^ eosinophils (*p* < 0.05). When comparing mice sensitised once and twice for the same cell types, the double-sensitised mice showed a tendency for higher percentages of positive cells, but the differences were not statically significant. No differences in the frequency of macrophages, Ly6G^+^ neutrophils, or CD19^+^ B cells were determined when comparing naïve, non-sensitised, and sensitised mice.

### Upregulated expression of genes encoding for Th2- and Th1-like cytokines in mice after sensitisation with peanut allergen

**Figure 3:**
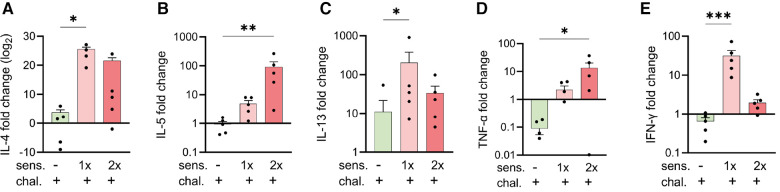
Expression of cytokine genes in peanut-allergen-treated mice via qPCR analysis. Lung tissue samples from mice described in Figure 1 were isolated, and gene expression patterns of cytokines were analysed by qPCR. Fold changes of IL-4 (**A**), IL-5 (**B**) IL-13 (**C**), TNF-α (**D**), and IFN-γ (**E**) over untreated mice were normalised to the housekeeping gene PPIA. Data are shown as mean ± SEM and analysed with the Kruskal–Wallis with Dunn multiple comparison test. *p < 0.05, **p < 0.01, ***p < 0.001. sens, sensitised.

To complement the immune analysis of lungs from peanut-allergen-sensitised mice challenged intranasally, we conducted qPCR analyses on lung tissue samples for quantification of the expression of mRNA encoding for cytokines (Figure 3). The samples from the sensitised and challenged mice were compared with those from challenged-only mice. For all the genes of interest, few to no changes were observed in the challenged-only mice. The sensitised mice exhibited an increased expression of the Th2-like and asthma-associated cytokines, IL-4 (Figure 3A), IL-5 (Figure 3B), and IL-13 (Figure 3C), as well as mRNA encoding for pro-inflammatory TNF-α (Figure 3D) and the Th1 cytokine IFN-γ (Figure 3E).

### Histological signs of inflammation in lung tissue of peanut-allergen-sensitised mice

**Figure 4:**
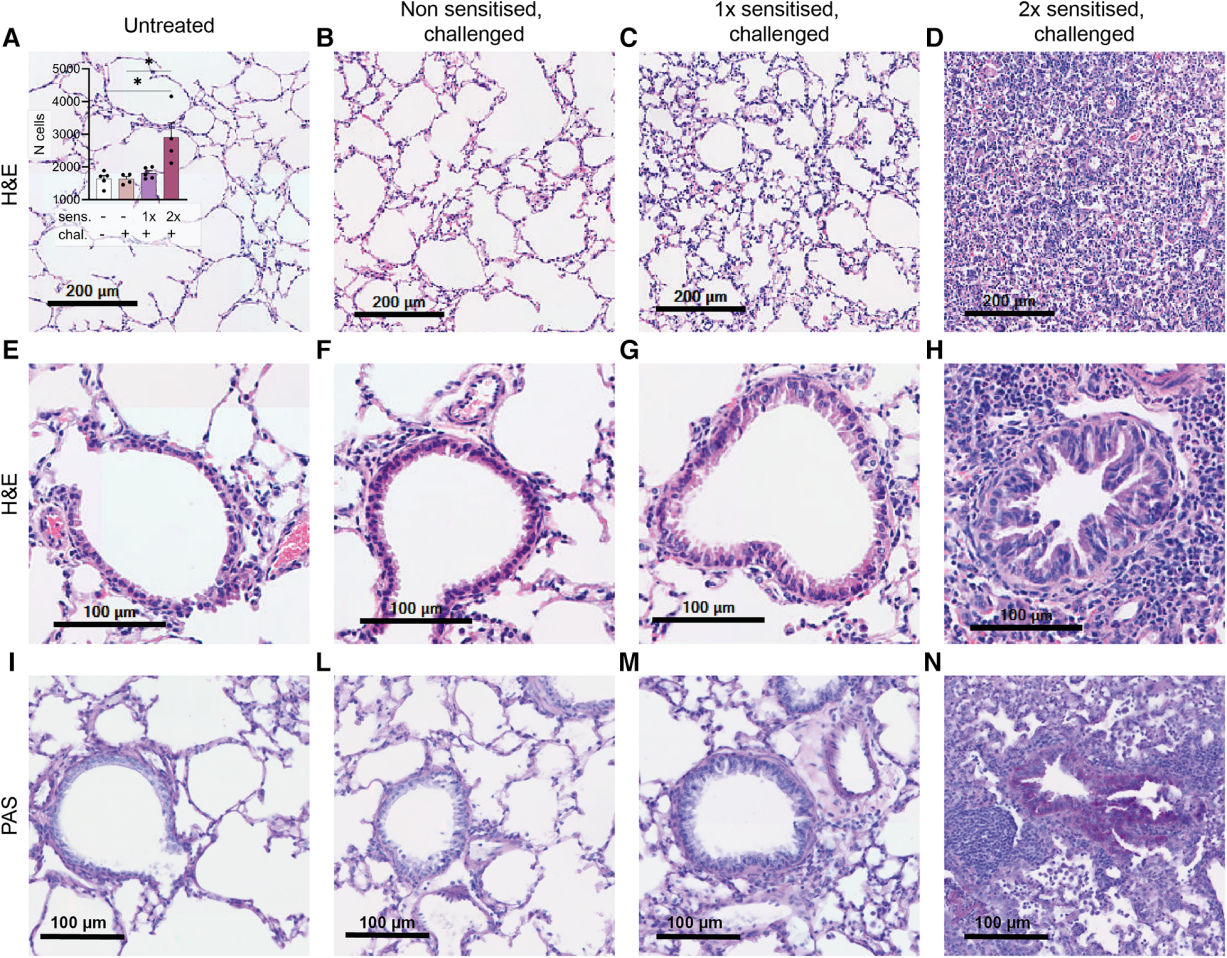
H&E and PAS staining of lung tissue sections. Lung tissue was obtained from mice that were treated as described in Figure 1. Representative H&E staining of alveoli (upper panel; **A**–**D**) with the relative cell counts (inset in **A**) and H&E and PAS staining of bronchioles (lower panels, **E**–**H** and **I**–**N**) are shown from untreated, challenged-only (non-sensitised, challenged), single-sensitised and challenged (1× sensitised, challenged), and double-sensitised and challenged mice (2× sensitised, challenged). Scale bar, 200 µm (upper panel) and 100 µm (lower panels). Data are shown as mean ± SEM and analysed with the Kruskal–Wallis with Dunn multiple comparison test. *p < 0.05.

One notable characteristic of asthma is the modification of lung tissue morphology caused by inflammation and the infiltration of immune cells, resulting in increased mucus production. Consequently, we assessed lung tissue sections from sensitised and challenged mice using H&E staining to examine cell infiltration, and PAS staining to identify carbohydrate macromolecules commonly present in the mucus. No signs of inflammation were observed in the lungs of non-sensitised untreated mice (Figure 4A) or in the lungs of non-sensitised mice that received an intranasal challenge with peanut allergen extract (Figure 4B). By contrast, lung tissue from mice sensitised once prior to the peanut allergen challenge exhibited inflammatory infiltrate (Figure 4C). However, when mice were sensitised with two intraperitoneal injections prior to the peanut allergen challenge, a much more severe allergic airway inflammation was observed (Figure 4D). In Figure 4A inset, the relative cell counts from lung tissue sections are presented. Statistical analysis revealed a significant difference in cell infiltration between non-sensitised mice with or without challenge and mice sensitised twice and challenged (*p* < 0.05). Further examination of the lung tissue (Figures 4E–H) revealed notable structural changes, including bronchial smooth muscle hypertrophy and mucus cells hyperplasia in addition to cellular infiltration in double-sensitised and challenged mice (Figure 4H). In addition, PAS staining (Figures 4I–N) showed increased numbers of PAS-positive areas in the airways of double-sensitised and challenged mice (Figure 4N), suggestive of increased mucus production. The immune cell infiltration was mostly localised around bronchioles and composed of macrophages with foamy multinucleated, giant foreign body cells. An accumulation of many eosinophilic granulocytes, few neutrophils, and very few lymphocytes was also noted. In addition, de-granulated mast cells appear to be present in sensitised lung tissue.

## Discussion

The classical acute model of allergic asthma in mice is based on ovalbumin (27, 28). While the ovalbumin model can reveal mechanisms involved in the pathogenesis of asthma and be useful for treatment targets of allergic inflammation (29), it is important also to include allergens that go beyond ovalbumin and that cause asthma in humans. Therefore, acute and chronic animal models have been developed with aeroallergens. However, patients with allergic sensitisation to foods such as peanut allergens are at risk for allergic inflammation of the airways (30, 31), and robust animal models of food allergy-associated asthma are needed. Therefore, this study describes an acute model of peanut-allergic airway inflammation in mice induced by IP sensitisation followed by intranasal allergen using a peanut allergen extract. This experimental setting of a systemic sensitisation phase and a pulmonary challenge phase is standard in IgE-mediated allergen-specific asthma in animals (32, 33). Our model demonstrated consistency with other murine models of allergy, in terms of allergen-specific IgE seroconversion, elevated Th2 cytokines, and remodelling of the airways, as manifested by structural changes such as bronchial smooth muscle hypertrophy, mucus cell hyperplasia, and infiltration of eosinophil cells. The latter is an important feature of asthma in humans (34–37).

While intranasal sensitisation would more resemble sensitisation in humans (38), we chose the IP route of administration as this has proven to require fewer doses, hence is fast, and since we have successfully used this route for the study of peanut-allergic anaphylaxis with the same allergen extract and for the same strain of mice (24, 39). Previous studies with other allergens often use two IP injections for sensitisation. Interestingly, we could show a single sensitisation was sufficient to trigger allergen-specific Th2 cytokine production, but that a second sensitisation was required to induce the remodelling of the lung tissue that is observed in asthma. A second sensitisation dose was also required to significantly raise the Ara-h-2-specific IgE levels, a further prerequisite of a mouse model of allergic asthma. When compared with the acute ovalbumin model of asthma (40), similar patterns of eosinophilic granulocyte accumulation in sensitised mice exposed to nasal allergen challenge were observed, both in BALF and in the lung tissue, further emphasising the role of eosinophils in driving allergic airway inflammation also in murine models of asthma (21, 41).

Our described model was also characterised by the involvement of T cells. The T-cell number in the BALF was increased in peanut-allergic mice with lung pathology. T-cell-derived cytokine production was determined in lung tissues as well as in lymphocytes after *in vitro* stimulation with peanut allergens, for instance by IL-2 release supportive of T-cell proliferation. Of interest, increased secretion of IFN-*γ* in mice subjected to nasal sensitisation and challenge was observed. However, this result aligns with other reports in murine models of peanut allergy (24, 42). Hence, the data suggest that the inflammatory response in this murine model might consist of an interplay of Th1- and Th2-associated processes (43, 44). To understand this underlying role of the Th1–Th2 interplay, further investigations would be required as the current study does not provide a definite explanation to that question. Although the standard Th1–Th2 paradigm remains a useful framework for understanding T-cell heterogeneity, different subsets of T cells may play different important roles in allergic asthma suggesting that asthma is not simply a Th2-type disease. Indeed, next to Th1 subsets, also Th17 cells, Th9 cells, and Tregs have been ascribed various roles in the pathology of asthma (45). Although the quantity of pulmonary B cells remained unchanged after nasal exposure to peanut allergens in our model, allergic B-cell responses may be ascribed a pivotal function in the production of allergen-specific IgE antibodies, rather than the proliferation and infiltration of B cells in lung tissue (46, 47).

Boyaka et al. were the first to describe lung reactivity in a mouse model of peanut allergy (42). In the work from 2005, the authors used a combined oral and nasal sensitisation regimen. Our model provides a cost-effective, simple, and fast method to establish peanut-allergic asthma in mice using a combined systemic and nasal sensitisation regimen, and the model may offer a valuable tool to study allergic asthma and potential novel therapeutic application for the treatment of peanut allergy. However, it is essential to acknowledge that our proposed model does not fully replicate the physiological sensitisation process in human peanut allergy. Therefore, recent efforts have been made to apply humanised mouse models in preclinical allergy (48) and asthma (49). Also the choice of the IP route for allergen exposure deviates from the typical gastrointestinal or epicutaneous routes for natural sensitisation (15, 50, 51). Furthermore, our study was limited to monitoring humoral and cellular immune responses and changes in gene expression. No conclusions can be drawn about the expected clinical effects like wheezing, dyspnoea, and reduced lung function (52, 53). Consequently, our murine model not only provides a partial understanding of the pathomechanisms underlying allergic asthma in humans, but also a starting point for development of a peanut-allergen-associated mouse model.

Given the lack of curative treatment options, the increasing prevalence, and a high socioeconomic burden associated with frequent emergency admissions, novel therapy options are urgently needed (54). Recently, oral immunotherapy (OIT) has been recommended as a potential treatment option for peanut allergy (55, 56). However, effectiveness was only seen in children and permanent desensitisation was not achieved (55). Patients already suffering from severe allergic airway inflammation undergoing OIT are at an increased risk of systemic allergic reactions, leaving an uncertainty regarding the safety of conventional immunotherapy (23, 55, 57, 58). Ongoing research is exploring novel therapeutic avenues. Passive allergen immunotherapy with monoclonal antibodies (mAbs) has demonstrated promising outlooks on reducing disease symptoms for birch- and cat- allergic patients (59, 60) and in mouse models of peanut allergy (39, 50, 61, 62). The development of additional mouse models that recapitulate different pathological aspects of peanut allergy, including complications linked to allergic asthma and airways inflammation, are therefore needed for therapeutic assessment. Our model, which effectively recapitulates airways inflammation, can be a valuable tool for researching and assessing the potential effects of novel therapeutic approaches. This model holds promise for making significant advancements in the pursuit of a cure for peanut-allergic asthma.

## Data Availability

The original contributions presented in the study are included in the article/Supplementary Material; further inquiries can be directed to the corresponding author.
